# The Climate Which Cures Consumption

**Published:** 1870-02

**Authors:** 


### THE CLIMATE WHICH CURES CONSUMPTION.

A series of valuable articles have appeared in the MEDICAL TIMES AND GAZETTE, on the climate of the Peruvian Andes.

We will suppose our readers acquainted, to some extent at least, with the recommendations which have been bestowed both on the Cordilleras of the Andes and on the plateaux of Mexico, of the cure of phthisis pulmonalis. In speaking of the last-named locality, Dr. Jourdanet strongly insists that this beneficial effect is altogether, or in greater part, due to elevation in soil, and not to the degree of latitude Dr. Smith, whose experience was gathered from Peru, expresses himself as follows :—"Incipient tuburcular phthisis, with more or less hæmoptysis, is one of the most common pulmonary affections known in Lima and other parts of the coast of Peru. * * It is a disease that is certainly cured by removing the patient from the coast to the open inland valley of Zanja, which runs from ten to twelve thousand feet above the sea level. * * This fact has been known and acted upon from time immemorial by the native inhabitants and physicians; and I have," says Dr. Smith, "sent patients from the capital to Zanja, in a very advanced state of phthisis, with open ulcerations and well-marked caverns in the lungs, and have seen them again, after the lapse of a little time, return to their homes free from fever, and with every appearance of the disease being arrested; but, in many instances, it would, after a protracted residence on the coast, again become necessary to return to the mountains, to prevent a recurrence of the disease." Dr. Scrivener expresses himself in the following terms :—"I have traversed these mountains on many occasions, and am therefore able to form an opinion of the salubrity of the climate, as also of that on the route from the Province of Cordova to the Pacific. All over this vast tract of land, that fatal enemy to man, the tubercular phthisis, so justly feared by the inhabitants of Lima and Buenos Ayres, is entirely unknown. During a residence of nearly ten years in different and widely spread districts of the whole country, I never saw nor heard, either directly or indirectly, through my intercourse with others, of the existence of that disease. In the mountains of Cordova, as well as on the Andine heights, the patient will find his disease alleviated and in time removed (let him come from what quarter of the globe he may), by the hand of nature. There pulmonary complaints are never known to originate, and there those who suffer from it, on the borders of the Parana and the river Plate, seek and find a permanent cure for their ailments, proceeding from all affections of the lungs. We would recommend," he further says, "the mountains of Cordova, to consumptive patients, in preference to the Andine heights of Bolivia, as being nearest the river Plate, and containing a greater variety of objects to divert the attention and amuse. The facility of transport, the shortness of the passage, combined with a well-founded hope of renovating the health, will be of themselves sufficient reasons for undertaking the journey. The mountains of Cordova are 4000 feet above the sea and about 800 miles from La Quiaca, the northern boundary of the Argentine Confederation, where commences the Bolivian territory; and here the Cordilleras of the Andes, or otherwise the Andine heights, are seen in all their splendor and magnificence. Between the two territories, however, the journey is made by mules; there is no carriage road.

It behooves us next to inquire how far and with what facility the heights of Cordova are accessible to patients proceeding thither from Europe. Let us suppose all of war at an end, the distant echoes of which are now heard brokenly on the road, and the journey already adventured on. The passage from England to Buenos Ayres may be made in as short a period as 34 days. There are several lines of merchant steamers from London and Liverpool, as well as the Government vessels from Southampton and Bordeaux, which arrive at Buenos Ayres every month. From this port you embark in a steamer for the port of Rosairo, which is most beautifully situated on the banks of the river Parana, and is the finest port in the Argentine Confederation, at which you arrive in about 26 hours. From thence you take the Argentine Central Railway, and arrive at the city of Cordova upon the same day. Here commence the serraicas or mountainous districts, which extend to the valley of Rimac, comprising an area of about 1000 leagues. We believe," says our author, "that at no distant time a public establishment will be founded in the mountains of Cordova, for use of consumptive patients. Should this be the case, we can vouch that there will be no lack of visitors willing to support the establishment and anxious to aid it by their means, in exchange for the benefits they have received there. The natural grandeur and magnificence of the mountain scenery would also contribute in no small degree to the attractions of the place and the benefit of the invalids. The city of Cordova is situated in a deep valley, on the banks of a river, amidst the most varied and beautiful scenery. Ascending from the city to the mountains, the traveller finds every variety of climate, with a difference of temperature at every ascent. In these varieties of temperature he will be certain to find one that is suitable to his complaint and agreeable to himself. The tops and sides of the mountains are covered with trees and shrubs, and the soil of the valleys is rich and very fertile, producing Indian corn, wheat, barley, sundry fruits and vegetables, and whatever the husbandman may desire to cultivate. Cattle, horses, mules, with sheep, roam in great herds on most excellent pasture. Huarracos and other wild animals inhabit the mountains. The wool of the sheep is of superior quality, and highly prized in European markets. There are a great variety of trees in the plains, many of which are very lofty, and their branches form an agreeable shade, as well as add to the beauty of the scenery. The timber of these trees is of superior quality, well suited for the construction of houses and in the manufacture of furniture, etc. There are mines of gold, silver, copper, and iron; the latter is abundant and in good quality. There are also marble quarries, and the marble is very fine and of different colors; limestone of an extremely white nature is abundant—in short, there are few spots in the world where Nature has lavished such a variety of animals, vegetables, and mineral productions as in the Province of Cordova.—Medical and Surgical Reporter.

